# Evolution of Orofacial Symptoms and Disease Progression in Idiopathic Parkinson's Disease: Longitudinal Data from the Jönköping Parkinson Registry

**DOI:** 10.1155/2017/7802819

**Published:** 2017-07-16

**Authors:** Seyed-Mohammad Fereshtehnejad, Örjan Skogar, Johan Lökk

**Affiliations:** ^1^Department of Neurology and Neurosurgery, McGill University, Montreal, QC, Canada; ^2^Division of Clinical Geriatrics, Department of Neurobiology, Care Sciences, and Society (NVS), Karolinska Institutet, Stockholm, Sweden; ^3^FUTURUM, Academy for Health and Care, Jönköping, Sweden; ^4^Department of Geriatric Medicine, Karolinska University Hospital, Stockholm, Sweden

## Abstract

**Background:**

Orofacial symptoms are common in Parkinson's disease (PD) both as initial manifestations and late markers of disease complications. We aimed to investigate the evolution of orofacial manifestations and their prognostic value throughout PD progression.

**Methods:**

Data was obtained from “Jönköping Parkinson Registry” database on routine care visits of 314 people with idiopathic PD in southern Sweden. Information on baseline symptomatology, orofacial features, UPDRS, and medications was recorded at baseline and during each follow-up visit within an average of 4.2 (range: 1–12) years.

**Results:**

Hypomimia, affected speech, drooling, and impaired swallowing were present in 37.3%/91.6%, 14.1%/65.5%, 11.7%/55.3%, and 10.2%/34.5% at baseline/follow-up, respectively. Male sex [OR = 2.4 (95% CI: 1.0–5.9)], UPDRS motor scores [OR = 1.2 (95% CI: 1.1–1.3)], dominant rigidity [OR = 5.2 (95% CI: 1.4–19.1)], and autonomic disturbance [OR = 3.4 (95% CI: 1.1–10.9)] were risk factors for drooling. Individuals with more severe orofacial burden at baseline had shorter median time to develop UPDRS-Part III > 28 [3rd tertile = 4.7 yr, 2nd tertile = 6.2 yr, and 1st tertile = 7.8 yr;* p* = 0.014].

**Conclusions:**

Majority of people with PD manifest orofacial manifestations at either early or late stages of the disease. PD severity, symmetry of motor disturbances, and autonomic disorders correlate with orofacial symptoms. Individuals with more severe orofacial burden at baseline progressed faster to more advanced stages.

## 1. Introduction

People with Parkinson's disease (PD) display a number of orofacial manifestations such as hypomimia, difficulties with speech, swallowing disturbances, and drooling of saliva. Cardinal motor features of PD, known as resting tremor, bradykinesia, and rigidity, could all potentially affect orofacial functions [1]. Neurophysiological studies show that, in PD, facial bradykinesia is primarily mediated by basal ganglia dysfunction [2], which reduces both the jaw mobility and the speed of jaw movements [3]. The process results in hypomimia, one of the most distinctive clinical features of PD. In parallel, rigidity and tremor complicate the formation and the placement of the food bolus as well as the chewing process [4, 5], which in turn induced food retention and dysphagia [1]. Drooling of saliva is another typical orofacial feature in PD caused by a combination of excessive spillover of saliva out of the oral cavity that brings various negative physical and psychosocial consequences for individuals with PD and their caregivers [6]. Several mechanisms including decreased frequency of swallowing, dysphagia, tongue tremor, diminished closure of the lips, and forward flexion of the head and neck known as antecollis contribute in the formation of sialorrhea in PD [7, 8]. 

Besides hypomimia as a clinical hallmark presented in almost all PD patients, other orofacial symptoms are also quite commonly occurred throughout the course of PD progression. Drooling and dysphagia, for instance, have been found in up to 78% [7] and 82% [9] of people with PD, respectively. Neuroepidemiologic studies have shown that orofacial manifestations can develop in mild to severe stages of PD [8] and their evolutionary trend varies from case to case. While in some individuals orofacial expressions represent early onset symptoms, for some others they happen in the late and potentially ominous states of PD. In addition to their psychosocial burden, orofacial dysfunctions, in particular dysphagia, was even once a major leading cause of mortality in PD through aspiration-induced pneumonia and choking in the pre-levodopa era [10]. Yet, due to the lack of true systematic prospective data collection in clinical practice, the knowledge in this field is insufficient. There is not enough comprehensive study on the evolution of orofacial dysfunctions and their contributing factors in PD. Furthermore, orofacial manifestations are usually easy to notice and feasible to track, which proposes them as suitable markers for disease progression.

Therefore, having access to a longitudinal cohort with rather long-term follow-up information, we aimed to investigate the following:

1. Prevalence of different orofacial symptoms throughout the course of PD;
2. Risk factors and determinants of different orofacial manifestations;
3. Prognostic value of the occurrence and severity of orofacial symptoms to predict progression in PD.

## 2. Methods

This prospective study was performed using data from an ongoing registry of people with PD recruited from the outpatient department of Jönköping referral hospital in southern Sweden, during 1998–2014.

### 2.1. Jönköping Parkinson Registry

Since the end of the late 90s, outpatient individuals with parkinsonism have been asked for and given their permission for registration in the “*Jönköping Parkinson Registry.”* This was one of the first attempts in Sweden to continuously register specified variables dealing with PD and parkinsonism-related disorders. The database was manufactured by the computer engineering department at the* County Hospital Ryhov*,* Jönköpings läns landsting*, a region with about 150000 inhabitants, in order to register diagnosis, actual medication, the current symptom information, and status of patients with PD and other forms of parkinsonian syndrome who attended the outpatient visits at the “Clinic of Medicine” and the “Clinic of Geriatrics.” Patients were primarily recruited from the primary care units of the region. After referral to the specialized clinics, they were routinely followed twice or at least once a year depending on the actual status of each individual. Data registration was performed during or immediately adjacent to each visit by the examining doctor. Some of the administrative data were collected and saved by the specialist nurse on duty. In total, five physicians, all with special interests in movement disorders, were involved in the registration of specific observations concerning orofacial symptoms.

The protocol for “*Jönköping Parkinson Registry”* and the access to the database was approved by the regional ethics committee. Participants were informed about the registration of their data and provided informed consent. Researchers followed all the sections, notes, and issues of the latest version of the Helsinki Declaration on research ethics. In order to ensure privacy, data were treated confidentially, protected from unauthorized access, and deidentified before analysis.

### 2.2. Study Population

In this study, we recruited 314 patients who fulfilled the clinical diagnosis of idiopathic Parkinson's disease (IPD) according to the UK Brain Bank Parkinson's Disease Criteria. Data from people with other parkinsonian syndromes were excluded.

### 2.3. Assessments

At baseline, demographic information, symptomatology, the Unified Parkinson's Disease Rating Scale (UPDRS) parts I–IV, and medications were recorded. A broad list of symptoms was assessed through self-reported answers or via basic physical examination by the attending neurologist at the clinic. The baseline symptoms consisted of resting tremor, bradykinesia, rigidity, fall, freezing, dyskinesia, wearing off, postural instability, visual impairment, hyposmia, urinary urgency, orthostatic hypotension, voice changes, and depression.

During each routine care visit, follow-up data consisting of UPDRS-Parts I–IV and change in antiparkinson drugs were also consecutively collected for each IPD patient over time. Follow-up data were available for 268 (85.4%) participants and the mean duration of follow-up time was 4.2 years ranging between >1 and 12 years. Using data from the UPDRS, scores were calculated for motor subtypes:

- *Tremor score*: mean of all UPDRS items on action and resting tremor on head and both left and right upper and lower extremities [11];
- *Postural instability–gait difficulty (PIGD) score*: sum of UPDRS-Part III items concerning rise, gait, and postural instability [12];
- *Asymmetry score*: absolute differences in UPDRS between left and right sides divided by the total UPDRS III (0 = perfect symmetry, 1 = absolute asymmetry) [13].

Orofacial manifestations were defined and assessed as follows:

- *Speech problems*: UPDRS-Part II-Item 5 score ≥2;
- *Drooling of saliva*: UPDRS-Part II-Item 6 score ≥2;
- *Swallowing difficulties*: UPDRS-Part II-Item 7 score ≥2;
- *Facial expression (hypomimia)*: UPDRS-Part III-Item 19 score ≥2.

Furthermore, a new aggregate indicator was obtained to represent the entire orofacial burden/severity by summing the scores on UPDRS items 5, 6, 7, and 19. For the assessment of disease progression, three outcome variables were defined as follows:

- *Progression in motor symptoms*: follow-up UPDRS-Part II total score >10 (above median)
- *Progression in motor signs*: follow-up UPDRS-Part III total score >28 (above median)

### 2.4. Statistical Analysis

Data were analyzed using SPSS software version 22 (IBM co., USA). To describe quantitative and qualitative variables mean [standard deviation (SD)] and frequency percentage (%) were reported, respectively. For univariate comparisons between the subgroups, Chi-square, Fisher's exact, and independent samples *t* tests were applied wherever appropriate. Multivariate binary logistic regression analyses were performed to investigate independent variables that associate with different orofacial symptoms after adjustment for sex, baseline age, duration of disease, and UPDRS-Part III (as the indicator of disease severity). For each orofacial manifestation, a multivariate regression model was created where, in addition to the list of control variables, other potential independent variables with a *p* value of <0.1 in univariate comparison were entered into the model. Multivariate Cox regression model was also applied to investigate the effects of baseline orofacial score on the progression of motor disability taking the time into account. Regarding the type of regression model, either odds' ratio (OR) or hazard ratio (HR) and their 95% confidence interval (CI) were reported.

Kaplan-Meier survival analysis was applied to calculate the mean and median time interval needed to develop certain outcomes of interest, namely, orofacial symptoms, UPDRS-Part I (>2), UPDRS-Part II (>10), and UPDRS-Part III (>28), of more than the median value of the maximum score during follow-up. In all analytical procedures, a two-tailed *p* value of less than 0.05 was considered to show statistical significant difference or association.

## 3. Results

### 3.1. Baseline Characteristics

A total number of 314 patients with IPD were recruited in this project. The study population consisted of 193 (61.5%) males and the average of age and disease duration was 64.7 (SD = 9.9) yr and 6.6 (SD = 5.5) yr at the time of registration. The average scores of the UPDRS-Part II, Part III, and entire scale at baseline were 9.3 (SD = 5.0), 21.7 (SD = 12.3), and 32.3 (SD = 15.0), respectively. More than 85% of the patients were under treatment with levodopa and dopamine agonists were prescribed in almost half of the study population. Other baseline and clinical characteristics are summarized in Table 1.

### 3.2. Prevalence and Time of Developing Orofacial Symptoms

**Figure 1:**
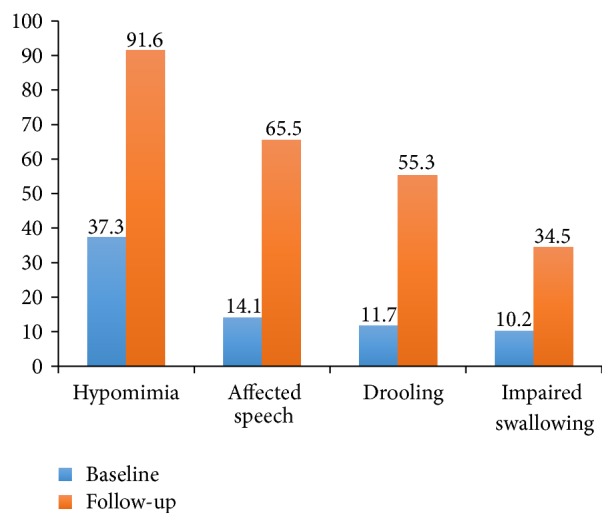
Prevalence rate (%) of different orofacial symptoms in study participants with idiopathic Parkinson's disease (IPD) at baseline and at the end of follow-up.

Hypomimia, affected speech, drooling, and impaired swallowing were present in 37.3%, 14.1%, 11.7%, and 10.2% of the IPD patients at baseline. More patients developed orofacial symptoms during the follow-up period. Hypomimia (91.6%) followed by speech problems (65.5%) and drooling (55.3%) were the most common orofacial symptoms in this cohort (Figure 1). Descriptive Kaplan-Meier survival analysis demonstrated that the average time to develop hypomimia and speech problems was 0.9 (SE = 0.1) yr and 6.4 (SE = 0.4) yr, respectively. It took longer time to develop considerable drooling [7.4 (SE = 0.4) yr] and swallowing difficulties [8.3 (SE = 0.4) yr] in IPD patients following the baseline assessment.

### 3.3. Determinant Factors of Orofacial Symptoms

Results from univariate comparison of baseline demographic and clinical characteristics between IPD patients with and without different orofacial symptoms are summarized in Table 2. At baseline, voice changes were more commonly presented in IPD patients who further developed speech problems (46.4% versus 9.3%, *p* < 0.001). Asymmetry index was significantly lower in this subgroup of participants as well as the ones with drooling, swallowing difficulties, and hypomimia (all *p* < 0.05). Orthostatic hypotension was more prevalent in patients with drooling (23.8% versus 6.6%, *p* = 0.044). IPD patients with prominent hypomimia had significantly higher prevalence of rigidity (83.0% versus 60.5%, *p* = 0.004), dyskinesia (30.0% versus 9.3%, *p* = 0.009), and urinary urgency (29.0% versus 4.7%, *p* = 0.001) compared to those who did not develop hypomimia (Table 2). As shown in Table 2, administration of different medications including levodopa, dopamine agonist, MAO-B inhibitor, and other dopaminergic drugs was not significantly different between IPD patients with and without orofacial symptoms (all *p* > 0.05). However, levodopa was in general more commonly used among those who developed orofacial symptoms (Table 2). We also analyzed whether medications correlate with the progression of orofacial symptoms over time. After adjusting by disease duration, administration of MAO-B inhibitor significantly lessened the progression of drooling over time (*p* = 0.011).

Multivariate analysis was performed to explore the factors that independently associated with the development of orofacial symptoms after adjustment for sex, baseline age, duration, and severity of the disease (measured by UPDRS-Part III). As shown in Table 3, except for swallowing difficulties, male sex was a risk factor for developing speech problems [OR = 2.3 (95% CI: 1.0–4.9)], drooling [OR = 2.4 (95% CI: 1.0–5.9)], and hypomimia [OR = 2.0 (95% CI: 1.1–3.6)]. More asymmetric manifestation [OR = 0.2 (95% CI: 0.1–0.8)] and higher tremor score [OR = 0.3 (95% CI: 0.1–0.7)] were found to be protective factors for speech impairment. Participants with dominant rigidity [OR = 5.2 (95% CI: 1.4–19.1)] and autonomic disturbances [OR = 3.4 (95% CI: 1.1–10.9)] at baseline were more likely to develop drooling. Urinary urgency was a strong determinant factor for considerable hypomimia during follow-up [OR = 6.7 (95% CI: 1.5–30.7)].

### 3.4. Prognostic Value of Orofacial Symptoms for Disease Progression

**Figure 2:**
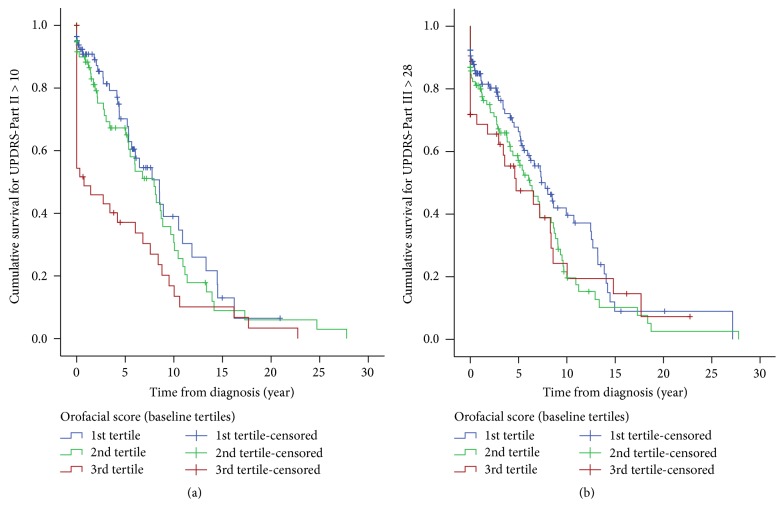
Survival curve to progress into the more than median value of the maximum UPDRS scores during the follow-up in patients with idiopathic Parkinson's disease with different severities of orofacial symptoms at baseline: (a) UPDRS-Part II: 1st tertile [median survival time = 8.5 yr (SE = 1.4)], 2nd tertile [median survival time = 8.0 yr (SE = 1.3)], and 3rd tertile [median survival time = 0.8 yr (SE = 0.6)] (Breslow *p* value < 0.001). (b) UPDRS-Part III: 1st tertile [median survival time = 7.8 yr (SE = 1.0)], 2nd tertile [median survival time = 6.2 yr (SE = 0.9)], and 3rd tertile [median survival time = 4.7 yr (SE = 2.2)] (Breslow *p* value = 0.014).

Kaplan-Meier analysis showed that IPD patients with speech impairments [4.4 (SE = 0.6) yr versus 8.5 (SE = 0.4) yr; *p* = 0.006] and drooling [5.0 (SE = 1.6) yr versus 8.1 (SE = 0.8) yr; *p* = 0.018] more quickly progressed to the UPDRS-Part II score of >10. In addition, participants with hypomimia [5.4 (SE = 0.6) yr versus 12.5 (SE = 3.2) yr; *p* = 0.001] and drooling [4.9 (SE = 1.0) yr versus 7.2 (SE = 0.9) yr; *p* = 0.030] had worse prognosis by more rapid progression to the UPDRS-Part III score of >28. As illustrated in Figure 2, further analysis was performed dividing study population into three tertiles based on the sum of the baseline UPDRS scores on orofacial items. IPD patients with more severe orofacial symptoms at baseline had a shorter median time to develop into a worse motor disability status showed by UPDRS-Part II > 10 [3rd tertile = 0.8 yr (SE = 0.6), 2nd tertile = 8.0 yr (SE = 1.3), and 1st tertile = 8.5 yr (SE = 1.4); *p* < 0.001] and UPDRS-Part III > 28 [3rd tertile = 4.7 (SE = 2.2) yr, 2nd tertile = 6.2 (SE = 0.9) yr, and 1st tertile = 7.8 (SE = 1.0) yr; *p* = 0.014]. Finally, results from a multivariate Cox regression model showed that baseline orofacial burden score [HR = 1.10 (95% CI: 1.01–1.20)] was a stronger predictor of hazard of developing UPDRS-Part III > 28 when compared to baseline PIGD score [1.08 (95% CI: 0.92–1.28)]. Similarly, baseline orofacial score [HR = 1.20 (95% CI: 1.10–1.31)] significantly associated with hazard of UPDRS-Part II > 10, whereas baseline PIGD score failed [HR = 0.75 (95% CI: 0.56–1.00)].

## 4. Discussion

Capitalizing upon the collection of longitudinal data from routine clinical practice of a PD cohort, our study is one of the few to extensively explore the picture of orofacial symptoms in people with IPD. Hypomimia was found to be the most common orofacial manifestation both at the baseline and throughout the course of progression followed, respectively, by speech problems, drooling of saliva, and impaired swallowing. More symmetric involvement, autonomic disturbances, and male gender were found to be significant determinants of developing different orofacial symptoms in PD. We also created a new single indicator to represent orofacial burden by adding up the scores of all orofacial manifestations. The indicator was significantly correlated to disease prognosis after an average of 4.2 years. The more severe orofacial burden was at baseline, the more rapidly individuals progressed into a more severe motor disability state. Interestingly, the single indicator, orofacial burden, showed more promising results in prediction of PD progression compared to the PIGD score at baseline.

Having gathered data on an orofacial screening test consisting of masticatory performance and oral health, Bakke et al. showed consistent correlation between the severity of PD (measured by Hoehn and Yahr and UPDRS score) and orofacial manifestations [1]. As also shown by our study, it is demonstrated that, in general, severity of orofacial symptoms is a reflection of the severity of motor disability in PD. In line with our results, another study showed that drooling of saliva more commonly occurred in males and those with a more advanced PD, though it could also happen as early as in Hoehn and Yahr stages 1 and 2, too [8, 14]. Kalf et al., in an investigation on 104 consecutive outpatients showed that people with PD who complained of drooling were significantly older and had longer disease duration and more severe PD [15]. While we also found higher UPDRS score as a significant determinant of drooling, disease duration and age failed to be strong associates of drooling in our cohort. The lack of a prominent association between disease duration and drooling has been recently shown by Ou et al. in another large cohort of 518 Chinese PD patients [16], too. However, this study also demonstrated higher UPDRS-Part-III as a significant determinant for drooling [16]. The fact that drooling is mostly associated with higher motor disability but not necessarily with disease duration indicates that drooling could be a manifestation of a more rapidly progressing phenotype of PD.

Our study is one of the few to report an association between autonomic dysfunction and more symmetric pattern with drooling. This connection was shown by few other studies [14, 17] and is consistent with the theory on the involvement of the vagus nerve in the pathophysiologic pathway of drooling via controlling deglutition and esophageal motility [18].

Impaired swallowing or dysphagia, as another common orofacial manifestation of PD, was present in 34.5% of our cohort during the follow-up. A recent systematic review revealed that a wide range of prevalence from 11% to 81% has been reported for dysphagia in different PD populations [19]. Our findings showed that swallowing ability is more likely to be impaired in individuals with longer duration of PD and a more symmetric phenotype. Another study found that dysphasia progression is closely associated with age at onset and severity of motor disability in PD [20]. Other determinants of dysphagia in PD reported in the literature are low body weight, drooling of saliva, and dementia [21]. Prevalence of speech problems increased by 4-5 times from baseline and reached up to 65% within the course of PD progression in our study. It has been estimated that as high as 89% of PD populations eventually experience speech impairment [22]. In line with our findings, Dias et al. also found that speech impairment does not correlate with the age of onset, but it is more likely to happen in people with more severe PD [23]. Moreover, according to our findings, speech problems were also more common among males, and those with a more symmetric PD. Our findings of higher burden of orofacial manifestations in people with more symmetric PD are consistent with a recent longitudinal investigation showing more symmetric distribution of motor symptoms in PD accompanies with a poorer prognosis in general [24]. Even though this association is thought to be entirely explained by the confounding effect of higher age and longer disease duration [24], our multivariate analysis showed that symmetric PD correlates with higher prevalence of orofacial features independent of disease duration and severity.

To our knowledge, our study is one of the few to comprehensively explore the evolution of orofacial symptoms in PD and the first to investigate the prognostic value of the aggregated orofacial burden for PD prognosis with longitudinal follow-up. Nevertheless, we acknowledge the limitations of our study. First, due to the real-world registry-based nature of our database and the time limitation during each visit, we did not have information on various other manifestations of PD such as cognition, sleep problems, full autonomic assessment, and psychiatric features. Second, based on the same reasons, we used subjective self-reported data for some symptoms (i.e., depression, hyposmia) and not validated batteries, questionnaires, and instruments. Yet, all examinations and assessments were done by expert neurologists and even self-reported data are quite commonly and reliably used in register-based settings. Lastly, detailed information on treatment options, in particular the specific interventions for drooling such as botulinum toxin, was not available.

In conclusion, orofacial manifestations are important components of PD phenotyping. Majority of people with PD evolve orofacial problems such as hypomimia, speech problems, drooling of saliva, and impaired swallowing. Measures of disease severity, symmetry, and autonomic disturbance correlate with orofacial manifestations. Individuals with more severe orofacial burden at baseline progressed faster to the advanced stages of PD with further motor disability. This prognostic value could be an applicable finding bearing in mind that orofacial manifestations are quite visible and easy to recognize. In addition, orofacial symptoms such as drooling and speech problems may potentially cause embarrassment, suffering, and increased disease burden [18]. Nevertheless, orofacial features of PD have not been fully addressed and are frequently neglected by the patients, their caregivers, and their clinicians. Greater awareness of the importance of orofacial features in PD is needed to optimize their management and potentially slow down the rate of disease progression.

From another perspective, our study highlights the usefulness of data registration in PD outpatient clinics. Having collected data from clinical practice, such real-world information could be a great reflection of what is routinely happening in people with PD, what is the natural course of progression, and what are its predictors. Of note, the “*Jönköping Parkinson Registry*” is now a part of the national PD register in Sweden launched in 2011.

## Figures and Tables

**Table 1:** Baseline, demographic, and clinical characteristics of the study population with idiopathic Parkinson's disease (IPD) (*n* = 314).

Characteristic	Value
Sex-male (%)	61.5%
Age (at baseline) (yr) mean (SD)	64.7 (9.9)
Disease duration (yr) mean (SD)	6.6 (5.5)
Follow-up time (yr) mean (SD)	3.5 (2.9)
Baseline symptoms (%)	
Resting tremor	76.9%
Bradykinesia	73.4%
Rigidity	76.2%
Fall	18.2%
Freezing	13.3%
Dyskinesia	23.8%
Wearing off	14.7%
Postural instability	8.4%
Visual impairment	19.6%
Hyposmia	9.1%
Urinary urgency	21.7%
Orthostatic hypotension	12.6%
Voice changes	16.8%
Depression	18.2%
UPDRS score (at baseline) mean (SD)	
Part I	0.8 (1.3)
Part II	9.3 (5.0)
Part III	21.7 (12.3)
Part IV	0.5 (1.7)
Total	32.3 (15.0)
Type of medication (%)	
Levodopa	85.3%
Dopamine agonist	49.2%
MAO-B inhibitor	13.2%
Other dopaminergics	5.6%
Orofacial symptoms (during follow-up) (%)	
Drooling	55.3%
Speech problems	65.5%
Swallowing difficulties	34.5%
Facial expression	91.6%

SD: standard deviation; UPDRS: Unified Parkinson's Disease Rating Scale.

**Table 2:** Univariate comparison of the baseline, demographic, and clinical characteristics of Parkinson's disease patients regarding orofacial symptoms.

Characteristic	Speech			Salivation			Swallowing			Facial expression		
	Normal (*n* = 141)	Affected (*n* = 65)	*p* value	Normal (*n* = 158)	Affected (*n* = 48)	*p* value	Normal (*n* = 170)	Affected (*n* = 36)	*p* value	Normal (*n* = 116)	Affected (*n* = 195)	*p* value
Sex-male (%)	60.3%	73.8%	0.059	62.0%	72.9%	0.167	62.9%	72.2%	0.290	55.2%	65.6%	0.066
Age (at baseline) (yr) mean (SD)	61.7 (8.7)	62.9 (10.0)	0.388	61.7 (9.1)	63.3 (9.2)	0.279	62.4 (8.8)	60.4 (10.4)	0.213	64.0 (10.6)	65.1 (9.6)	0.359
Disease duration (yr) mean (SD)	7.0 (5.7)	7.6 (5.7)	0.556	7.1 (5.8)	7.4 (5.5)	0.768	6.7 (5.5)	9.2 (5.8)	**0.032**	5.8 (5.6)	7.0 (5.4)	0.144
Baseline symptoms (%)												
Resting tremor	79.6%	67.9%	0.239	77.0%	71.4%	0.605	73.1%	86.7%	0.339	86.0%	73.0%	0.090
Bradykinesia	81.5%	75.0%	0.492	75.4%	90.5%	0.214	83.6%	60.0%	**0.042**	62.8%	78.0%	0.059
Rigidity	77.8%	85.7%	0.559	75.4%	95.2%	0.058	80.6%	80.0%	1	60.5%	83.0%	**0.004**
Fall	20.4%	14.3%	0.563	16.4%	23.8%	0.448	14.9%	33.3%	0.096	18.6%	18.0%	0.931
Freezing	16.7%	14.3%	1	18.0%	9.5%	0.498	16.4%	13.3%	1	7.0%	16.0%	0.185
Dyskinesia	31.5%	28.6%	0.786	34.4%	19.0%	0.273	26.9%	46.7%	0.132	9.3%	30.0%	**0.009**
Wearing off	24.1%	10.7%	0.239	21.3%	14.3%	0.750	16.4%	33.3%	0.135	9.3%	17.0%	0.307
Postural instability	7.4%	10.7%	0.686	8.2%	9.5%	1	6.0%	20.0%	0.111	4.7%	10.0%	0.511
Visual impairment	31.5%	17.9%	0.187	27.9%	23.8%	0.717	28.4%	20.0%	0.748	11.6%	23.0%	0.116
Hyposmia	13.0%	10.7%	1	9.8%	19.0%	0.269	13.4%	6.7%	0.680	9.3%	9.0%	0.954
Urinary urgency	20.4%	28.6%	0.404	18.0%	38.1%	0.060	25.4%	13.3%	0.501	4.7%	29.0%	**0.001**
Orthostatic hypotension	7.4%	17.9%	0.262	6.6%	23.8%	**0.044**	10.4%	13.3%	0.666	11.6%	13.0%	0.821
Voice changes	9.3%	46.4%	**<0.001**	21.3%	23.8%	0.811	20.9%	26.7%	0.731	9.3%	20.0%	0.146
Depression	16.7%	21.4%	0.597	18.0%	19.0%	1	17.9%	20.0%	1	14.0%	20.0%	0.390
UPDRS score (at baseline) mean (SD)												
Part I	1.1 (1.5)	1.0 (1.4)	0.558	1.1 (1.5)	1.2 (1.6)	0.756	1.1 (1.5)	1.1 (1.6)	0.890	0.6 (1.1)	0.9 (1.5)	**0.028**
Part II	8.6 (5.1)	11.0 (4.3)	**0.011**	8.4 (4.3)	12.2 (5.9)	**<0.001**	9.2 (5.2)	9.6 (4.1)	0.735	7.4 (3.8)	9.9 (4.5)	**0.002**
Part III	18.6 (9.5)	22.0 (10.9)	**0.027**	18.2 (9.5)	24.4 (10.3)	**<0.001**	19.2 (9.7)	21.5 (11.4)	0.212	17.3 (10.8)	24.4 (12.4)	**<0.001**
Part IV	0.7 (1.9)	0.7 (1.9)	0.801	0.7 (1.9)	0.8 (2.1)	0.840	0.8 (2.0)	0.6 (1.3)	0.633	0.2 (1.1)	0.7 (1.9)	**0.013**
Total	30.1 (14.2)	37.9 (15.9)	**0.010**	29.4 (13.8)	41.1 (15.5)	**<0.001**	31.9 (14.4)	34.0 (17.8)	0.567	25.2 (13.4)	35.9 (14.6)	**<0.001**
Type of medication (%)												
Levodopa	78.8%	90.9%	0.137	81.4%	87.5%	0.590	80.8%	92.0%	0.243	78.0%	88.4%	0.058
Dopamine agonist	49.4%	61.4%	0.197	55.7%	46.9%	0.387	54.8%	48.0%	0.540	54.2%	47.1%	0.359
MAO-B inhibitor	15.3%	9.1%	0.416	11.3%	18.8%	0.283	13.5%	12.0%	1	10.2%	14.5%	0.412
Other dopaminergics	7.1%	4.5%	0.715	6.2%	6.3%	1	6.7%	4.0%	1	5.1%	5.8%	1
Disease parameters mean (SD)												
Tremor score	0.5 (0.5)	0.4 (0.4)	0.063	0.5 (0.4)	0.5 (0.5)	0.731	0.5 (0.4)	0.6 (0.5)	0.296	0.4 (0.4)	0.5 (0.5)	0.072
PIGD score	0.6 (0.6)	0.7 (0.5)	0.207	0.6 (0.5)	0.8 (0.7)	0.069	0.6 (0.6)	0.6 (0.6)	0.728	0.7 (0.7)	0.8 (0.8)	0.203
Asymmetry index	0.6 (0.4)	0.4 (0.3)	**0.001**	0.6 (0.4)	0.4 (0.3)	**0.004**	0.6 (0.4)	0.4 (0.3)	**0.035**	0.6 (0.4)	0.4 (0.3)	**<0.001**

SD: standard deviation; UPDRS: Unified Parkinson's Disease Rating Scale; PIGD: postural instability/gait difficulty; all orofacial symptoms were considered present if a score of at least 2 in the UPDRS-Part II or III was recorded at any time during the follow-up period.

**Table 3:** Multivariate regression models for significant independent determinant factors of different orofacial symptoms in patients with idiopathic Parkinson's disease (IPD).

Orofacial symptom	Significant determinants *(Logistic regression model)*
Speech	Male sex: OR = 2.3 (95% CI: 1.0–4.9)
	UPDRS-Part III: OR = 1.1 (95% CI: 1.0–1.1)
	Voice changes: OR = 9.4 (95% CI: 2.6–33.2)
	Tremor score: OR = 0.3 (95% CI: 0.1–0.7)
	Asymmetry index: OR = 0.2 (95% CI: 0.1–0.8)

Salivation	Male sex: OR = 2.4 (95% CI: 1.0–5.9)
	UPDRS-Part II: OR = 1.2 (95% CI: 1.1–1.3)
	UPDRS-Part III: OR = 1.1 (95% CI: 1.0–1.1)
	Dominant rigidity: OR = 5.2 (95% CI: 1.4–19.1)
	Autonomic disturbance: OR = 3.4 (95% CI: 1.1–10.9)

Swallowing	Disease duration: OR = 1.1 (95% CI: 1.0–1.2)

Facial expression	Male sex: OR = 2.0 (95% CI: 1.1–3.6)
	UPDRS-Part III: OR = 1.1 (95% CI: 1.0–1.1)
	Dominant rigidity: OR = 2.8 (95% CI: 1.1–6.6)
	Dyskinesia: OR = 5.7 (95% CI: 1.6–19.8)
	Urinary urgency: OR = 6.7 (95% CI: 1.5–30.7)

OR: odds' ratio; UPDRS: Unified Parkinson's Disease Rating Scale; all orofacial symptoms were considered present if a score of at least 2 in the UPDRS-Part II or III was recorded at any time during the follow-up period; all variables with a *p* value of <0.1 in univariate comparisons were checked for an independent effect in multivariate regression models; all regression models have been adjusted at least for sex, baseline age, duration of disease, and UPDRS-Part III (as the indicator of disease severity either as score or tertile); all presented determinant factors showed a *p* value of <0.05 in each multivariate regression model.

## References

[1] Bakke M., Larsen S. L., Lautrup C., Karlsborg M. (2011). Orofacial function and oral health in patients with Parkinson's disease. *European Journal of Oral Sciences*.

[2] Bologna M., Fabbrini G., Marsili L., Defazio G., Thompson P. D., Berardelli A. (2013). Facial bradykinesia. *Journal of Neurology, Neurosurgery and Psychiatry*.

[3] Robertson L. T., Hammerstad J. P. (1996). Jaw movement dysfunction related to Parkinson's disease and partially modified by levodopa. *Journal of Neurology Neurosurgery and Psychiatry*.

[4] Karlsson S., Persson M., Johnels B. (1992). Levodopa induced on-off motor fluctuations in Parkinson's disease related to rhythmical masticatory jaw movements. *Journal of Neurology Neurosurgery and Psychiatry*.

[5] Leopold N. A., Kagel M. C. (1996). Prepharyngeal dysphagia in Parkinson's disease. *Dysphagia*.

[6] Srivanitchapoom P., Pandey S., Hallett M. (2014). Drooling in Parkinson's disease: a review. *Parkinsonism and Related Disorders*.

[7] Chou K. L., Evatt M., Hinson V., Kompoliti K. (2007). Sialorrhea in Parkinson's disease: a review. *Movement Disorders*.

[8] Rana A. Q., Yousuf M. S., Awan N., Fattah A. (2012). Impact of progression of Parkinson's disease on drooling in various ethnic groups. *European Neurology*.

[9] Kalf J. G., de Swart B. J. M., Bloem B. R., Munneke M. (2012). Prevalence of oropharyngeal dysphagia in Parkinson's disease: a meta-analysis. *Parkinsonism and Related Disorders*.

[10] Sutton J. P. (2013). Dysphagia in Parkinson's disease is responsive to levodopa. *Parkinsonism and Related Disorders*.

[11] Schiess M. C., Zheng H., Soukup V. M., Bonnen J. G., Nauta H. J. W. (2000). Parkinson's disease subtypes: Clinical classification and ventricular cerebrospinal fluid analysis. *Parkinsonism and Related Disorders*.

[12] Van Rooden S. M., Colas F., Martínez-Martín P. (2011). Clinical subtypes of Parkinson's disease. *Movement Disorders*.

[13] Espay A. J., Li J.-Y., Johnston L., Chen R., Lang A. E. (2005). Mirror movements in parkinsonism: Evaluation of a new clinical sign. *Journal of Neurology, Neurosurgery and Psychiatry*.

[14] Verbaan D., Marinus J., Visser M., Van Rooden S. M., Stiggelbout A. M., Van Hilten J. J. (2007). Patient-reported autonomic symptoms in Parkinson disease. *Neurology*.

[15] Kalf J. G., Bloem B. R., Munneke M. (2012). Diurnal and nocturnal drooling in Parkinson's disease. *Journal of Neurology*.

[16] Ou R., Guo X., Wei Q. (2015). Prevalence and clinical correlates of drooling in Parkinson disease: A study on 518 Chinese patients. *Parkinsonism and Related Disorders*.

[17] Siddiqui M. F., Rast S., Lynn M. J., Auchus A. P., Pfeiffer R. F. (2002). Autonomic dysfunction in Parkinson's disease: a comprehensive symptom survey. *Parkinsonism and Related Disorders*.

[18] Zlotnik Y., Balash Y., Korczyn A. D., Giladi N., Gurevich T. (2015). Disorders of the oral cavity in parkinson's disease and parkinsonian syndromes. *Parkinson's Disease*.

[19] Takizawa C., Gemmell E., Kenworthy J., Speyer R. (2016). A Systematic Review of the Prevalence of Oropharyngeal Dysphagia in Stroke, Parkinson’s Disease, Alzheimer’s Disease, Head Injury, and Pneumonia. *Dysphagia*.

[20] Liu L., Luo X.-G., Dy C.-L. (2015). Characteristics of language impairment in Parkinson's disease and its influencing factors. *Translational Neurodegeneration*.

[21] Suttrup I., Warnecke T. (2016). Dysphagia in Parkinson’s Disease. *Dysphagia*.

[22] Duffy J. R. (2005). *Motor Speech Disorders: Substrates, Differential Diagnosis, and Management*.

[23] Dias A. E., Barbosa M. T., Limongi J. C. P., Barbosa E. R. (2016). Speech disorders did not correlate with age at onset of Parkinson’s disease. *Arquivos de Neuro-Psiquiatria*.

[24] Marinus J., van Hilten J. J. (2015). The significance of motor (A)symmetry in Parkinson's disease. *Movement Disorders*.

